# Phosphatase of Regenerating Liver-1 (PRL-1) Regulates Actin Dynamics During Immunological Synapse Assembly and T Cell Effector Function

**DOI:** 10.3389/fimmu.2018.02655

**Published:** 2018-11-20

**Authors:** Patricia Castro-Sánchez, Rocío Ramirez-Munoz, Noa B. Martín-Cófreces, Oscar Aguilar-Sopeña, Sergio Alegre-Gomez, Sara Hernández-Pérez, Raquel Reyes, Qi Zeng, Carlos Cabañas, Francisco Sánchez-Madrid, Pedro Roda-Navarro

**Affiliations:** ^1^Department of Immunology, School of Medicine, Universidad Complutense de Madrid, Madrid, Spain; ^2^12 de Octubre Health Research Institute (imas12), Madrid, Spain; ^3^Servicio de Inmunología. Hospital Universitario de la Princesa, Universidad Autónoma de Madrid, Instituto de Investigación Sanitaria Princesa (IP), Madrid, Spain; ^4^Centro de Investigación Biomédica en Red de Enfermedades Cardiovasculares (CIBERCV), Madrid, Spain; ^5^Department of Cell Biology and Immunology, Center for Molecular Biology Severo Ochoa (CBM-SO), Mayor Council of Scientific Research (CSIC), Madrid, Spain; ^6^Institute of Molecular and Cell Biology, Agency for Science, Technology and Research (ASTAR), Singapore, Singapore

**Keywords:** T cell immune response, immunological synapse, phosphatases of regenerating liver, actin cytoskeleton, IL-2

## Abstract

The regulatory role of most dual specific phosphatases during T cell activation remains unknown. Here, we have studied the expression and function of phosphatases of regenerating liver (PRLs: PRL-1, PRL-2, and PRL-3) during T cell activation, as well as, the dynamic delivery of PRL-1 to the Immunological Synapse (IS). We found that T cell activation downregulates the expression of PRL-2, resulting in an increased PRL-1/PRL-2 ratio. PRL-1 redistributed at the IS in two stages: Initially, it was transiently accumulated at scanning membranes enriched in CD3 and actin, and at later times, it was delivered at the contact site from pericentriolar, CD3ζ-containing, vesicles. Once at the established IS, PRL-1 distributed to LFA-1 and CD3ε sites. Remarkably, PRL-1 was found to regulate actin dynamics during IS assembly and the secretion of IL-2. Moreover, pharmacological inhibition of the catalytic activity of the three PRLs reduced the secretion of IL-2. These results provide evidence indicating a regulatory role of PRL-1 during IS assembly and highlight the involvement of PRLs in immune responses by mature T cells.

## Introduction

Antigen-induced activation of T lymphocytes is mediated by the formation of the immunological synapse (IS), a dynamic supra-molecular structure organized at the interface of interacting T lymphocytes and antigen presenting cells (APCs) (1). The intracellular signaling downstream the T-cell receptor (TCR) triggers actin rearrangements, as well as, microtubule organizing center (MTOC) and endosomal compartment polarization to the IS. In turn, this cytoskeleton and endosomal compartment dynamics is critical for IS organization, sustains intracellular signaling and mediates cytokine secretion (2–5). Nonetheless the interplay between signaling networks and the cytoskeleton/endosomal compartment dynamics is not completely understood.

Phospho-tyrosine phosphatases (PTPs) can be classified as classical PTPs, which are specific for pTyr residues, and dual specificity phosphatases (DSPs), which are also specific for pSer, pThr, phospholipids, or nucleic acids (6, 7). Most data about regulatory functions of these enzymes in T cell activation refer to classical PTPs, including enzymes that dephosphorylate cytoskeleton regulators or signaling molecules (8–10). By contrast, the function of DSPs in T cell activation is mostly unknown, except for some enzymes that regulate the intracellular signaling by MAPK or phospholipids (8, 11, 12). In addition, the delivery of DSPs to the IS and their regulatory role in the dynamics of the cytoskeleton and the endosomal compartment during T cell immune responses have been barely studied (13, 14).

Phosphatases of regenerating liver (PRLs) comprise a group of 3 highly homologous small DSPs (PRL-1/*PTP4A1*, PRL-2/*PTP4A2*, and PRL-3/*PTP4A3*) overexpressed in several cancer types, including hematological malignancies (15). In the carboxyl terminal region, downstream the PTP catalytic domain, these DSPs contain a polybasic sequence and a CAAX motif for farnesylation that mediate the binding to cell membranes (16). *In situ* hybridization of human tissue specimens indicates a strong expression of genes coding for PRL-1 and PRL-2 in the T cell area of lymph nodes (17). Furthermore, PRL-1 has been previously proposed to regulate the actin cytoskeleton in tumor cells (18). These data suggest a regulatory role of PRLs in immune responses by T cells. Thus, we aim to study whether PRLs have a regulatory role during CD4 T cell activation. Here, we have evaluated the expression of PRLs in human primary CD4 T cells and tracked the dynamic delivery of PRL-1 at the IS. We have studied the regulatory role of this enzyme in actin dynamics occurring during T cell activation. Finally, we have assessed the production of IL-2 upon pharmacological inhibition of the catalytic activity of PRL-1 and of all PRLs. The obtained results suggest a regulatory role of PRLs during T cell immune responses.

## Results

### Expression of PRLs in human mature CD4 T cells

**Figure 1:**
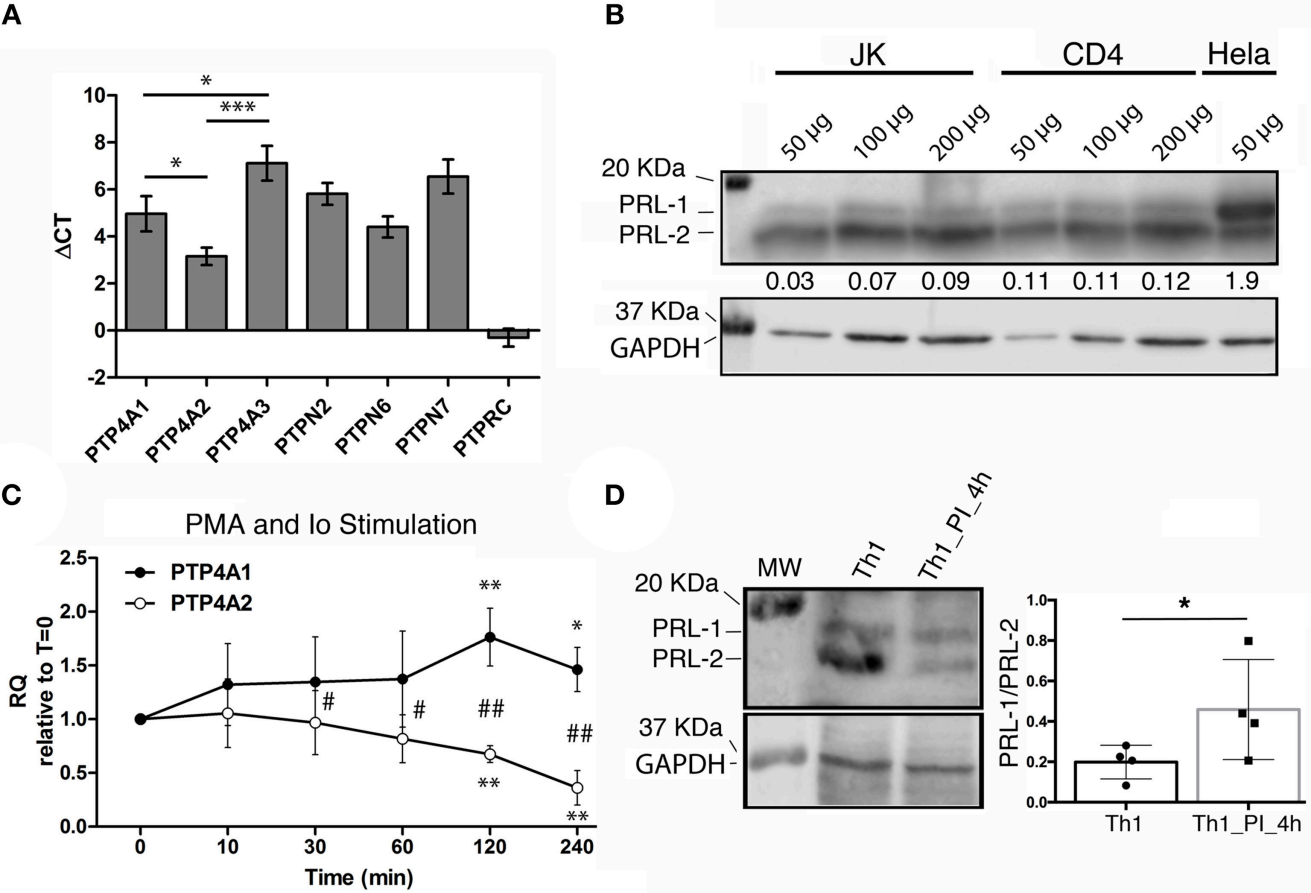
Expression of PRLs in mature CD4 T cells. **(A)** The gene expression of PRLs and other PTPs in peripheral blood CD4 T cells from *n* = 3 donors was analyzed by qPCR. The mean value of the ΔCT and the standard deviation (SD) for each gene is shown. Data of PRLs were compared by a one-way ANOVA. Asterisks indicate the *p*-value: ^*^P ≤ 0.05, ^***^P ≤ 0.001. **(B)** Western Blot for PRL-1 and PRL-2 detection in the CD4 T cell line Jurkat (JK), in peripheral blood CD4 T cells (CD4) and in the Hela cell line. The amount of protein loaded is indicated. Numbers under the PRL-1/PRL-2 blot indicate the normalized densitometry of PRL-1 vs. PRL-2. The molecular weight (MW) markers are indicated. One representative experiment is shown. **(C)** Expression of *PTP4A1* and *PTP4A2* mRNA in Th1 effectors upon stimulation with PMA and Ionomycin for the indicated times in minutes (min). Graphs represent the relative expression (RQ) with respect to time cero (*t* = 0). The mean ± SD is shown of RQ values from *n* = 4 different donors. Asterisks indicate the *p*-value of a one-sample *t*-test comparing each time to *t* = 0. Hashes indicate the *p*-value of a *t*-test comparing *PTP4A1* and *PTP4A2* expression at each time. ^*^ and ^#^*P* ≤ 0.05, ^**^ and ^##^*P* ≤ 0.01. **(D)** Western blot for PRL-1 and PRL-2 (upper left panel) and GAPDH (lower left panel) detection. The MW markers are indicated. Right panel shows the PRL-1/PRL-2 ratio. PI indicates PMA and Ionomycin stimulation. The graph shows the mean ± SD obtained from *n* = 4 donors analyzed. The mean of the sample was compared by a paired *t*-test. The asterisk indicates the *p*-value: ^*^P ≤ 0.05.

The reported strong expression of *PTP4A1* and *PTP4A2* in the T cell area of lymph nodes (17) prompted us to evaluate the expression of the genes coding for PRLs in peripheral blood CD4 T lymphocytes. mRNA levels of *PTP4A1, PTP4A2*, and *PTP4A3* were similar to those of other genes coding for classical PTPs that regulate T cell immune responses, such as TC-PTP/*PTPN2*, SHP1/*PTPN6*, or HePTP/*PTPN7* (8) (Figure 1A). Among the group of PRLs, gene expression of *PTP4A2* was higher than those of *PTP4A1* and *PTP4A3* (Figure 1A). Protein levels of PRL-1 and PRL-2 in peripheral blood CD4 T lymphocytes and the CD4 T cell line Jurkat (JK) were consistent with mRNA levels (Figure 1B). Hela cells were used as control of PRL-1 and PRL-2 expression. Typical electrophoretic migration of PRL-1 and PRL-2 was found (19).

In order to gain insight about the function of these molecules during T cell activation, we studied the regulation of mRNA expression levels of *PTP4A1, PTP4A2*, and *PTP4A3* in *in vitro* expanded Th1 cells re-stimulated with phorbol esters and Iomomycin (PI treatment). PKC activation and intracellular rise of Ca^2+^ obtained by this treatment upregulated and downmodulated *PTP4A1* and *PTP4A2*, respectively (Figure 1C). At protein level, although an upregulation of PRL-1 was not detected, the PI treatment clearly downmodulated the expression of PRL-2, causing an increment in the relative amount of PRL-1 regarding PRL-2 (Figure 1D). A similar mRNA regulation of *PTP4A1* and *PTP4A2*, but not *PTP4A3*, was found in peripheral blood CD4 T cells (Supplementary Figures 1A,B). The regulation of *PTP4A1* and *PTP4A2* mRNA levels accompanied the induction of CD69 activation marker (Supplementary Figure 1C). These data indicated that T cell stimulation leading to PKC activation and Ca^2+^ elevation increased the relative amount of PRL-1 compared with PRL-2.

### PRL-1 localizes at the immunological synapse

**Figure 2:**
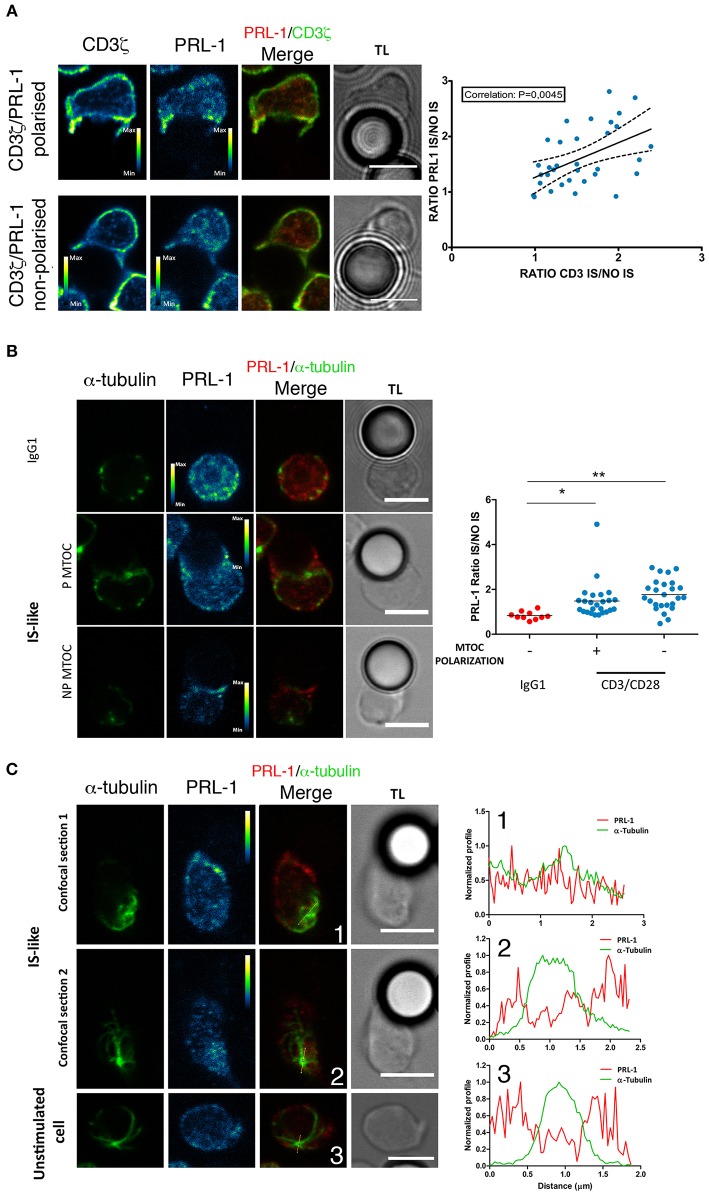
Distribution of endogenous PRL-1 at the IS. Representative images are shown of immunofluorescence of CD4 T cells interacting for up to 20 min (see material and methods) with microspheres coated with anti-CD3ε and anti-CD28 antibodies (IS-like interactions) or IgG1 as negative control for stimulation. Images represent confocal sections in the green and red channels, as well as, the merged of channels and the transmission light (TL). Calibration bar is shown in pseudocolored images. Scale bars 5 μm. **(A)** Examples of conjugates showing or not accumulation of PRL-1 and CD3ζ at the IS. The right panel represent the correlation of PRL-1 and CD3ζ accumulation at the IS. Dots represent individual conjugates. The *p*-value of the Pearson coefficient is shown. **(B)** Examples of conjugates showing or not accumulation of PRL-1 and the MTOC at the IS. The right panel represents the quantification of PRL-1 accumulation to the IS in relation to MTOC polarization. Dots represent individual conjugates. Groups were compared by a one-way ANOVA. Asterisks indicate the *p*-value: ^*^P ≤ 0.05, ^**^P ≤ 0.01. **(C)** Two confocal sections are shown of a cell forming an IS-like interaction and one confocal section of a non-stimulated cell. Right panels represent profiles of the fluorescence intensity in the green and the red channel along lines drawn in images. Numbers indicate the correspondence between the cell and the profile. (**A**–**C)** Data obtained from 2 experiments done with 2 different donors.

We then decided to investigate whether PRL-1 regulated the assembly of the IS and T cell effector functions. The distribution of PRL-1 at the IS was initially imaged in IS-like structures formed by peripheral blood CD4 T cells of healthy donors and micro-spheres coated with anti-CD3ε and anti-CD28 antibodies as previously described (20). Co-staining with antibodies specific for PRL-1 (Supplementary Figures 2A,B) and CD3ζ revealed a correlation in the polarization of both molecules at IS-like structures (Figure 2A), suggesting that PRL-1 enrichment was associated to the activation process. Co-staining with antibodies specific for alpha tubulin revealed the accumulation of PRL-1 at the IS-like structures in cells showing or not polarization of the microtubule-organizing center (MTOC), while it was not recruited to interfaces established with non-stimulating control beads (Figure 2B). Interestingly, two pools of PRL-1 were observed; one around the MTOC and other accumulated at the IS (Figure 2C).

**Figure 3:**
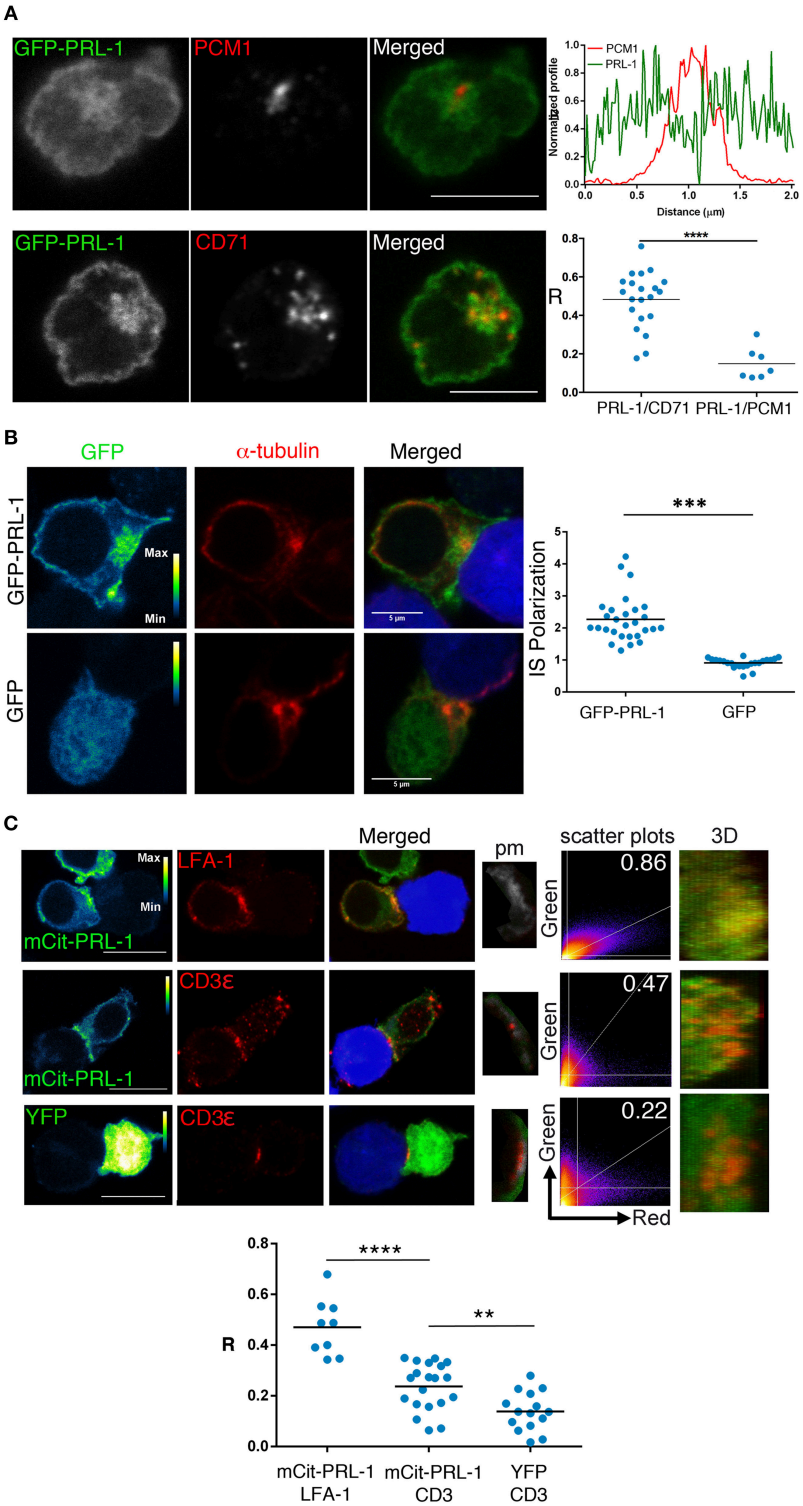
Subcellular distribution of PRL-1 in JK cells. **(A)** Representative cells of Immunofluorescence experiments of JK cells overexpressing GFP-PRL-1 and stained for the pericentriolar material-1 (PCM1) or CD71. The upper and right panel represents the profile of the fluorescence intensities in the green and the red channel along the line drawn in the PRL-1/PCM1 merged image. Lower and right panel showed the Pearson's coefficient for quantifying the PRL-1 co-localization with CD71 or PCM1. Spots represent cells analyzed from *n* = 3 experiments. **(B)** Representative immunofluorescence of cell conjugates formed by JK cells overexpressing GFP-PRL-1 or GFP alone and RAJI cells labeled with CMAC (blue) and loaded with SEE. The right panel represents the quantification of the accumulation of GFP-PRL-1 or GFP alone at the IS. Dots represent individual conjugates obtained from *n* = 2 experiments. Groups were compared by a *t*-test. Asterisks indicate the *p*-value: ^***^p ≤ 0.001 **(C)** Immunofluorescence of cell conjugates formed by JK cells overexpressing mCit-PRL-1 or YFP alone and conjugated with RAJI cells loaded with SEE and labeled with CMAC (blue). The IS markers are shown in red. mCit-PRL-1 and YFP are shown as a pseudocolor image with the calibration bar. Co-localization is shown in a pixel map (pm) obtained at the interaction site. White pixels indicate co-localization sites. Scatter plots of green and red channels along the stack are shown. Numbers indicate Manders coefficients (MC). The interface surface obtained from a 3D reconstruction of the IS where co-localization was analyzed is shown. Scale bars 10 μm. Lower graph: Quantification of the co-localization by Pearson coefficients (R). Dots represent individual cell conjugates obtained from *n* = 2 (LFA-1 vs. PRL-1), *n* = 7 (CD3 vs. PRL-1) or *n* = 5 (CD3 vs. GFP) experiments. The different samples were compared by a *t*-test. Asterisks represent the *p*-values: ^**^P ≤ 0.01, ^****^P ≤ 0.0001.

We also studied by confocal microscopy the spatial distribution of PRL-1 in the CD4 T cell line Jurkat (JK) transfected with PRL-1 fluorescent fusion proteins (Supplementary Figure 2). Cells were imaged before and after the assembly of the mature IS established with Staphylococcal Enterotoxin E (SEE)-loaded Raji B cells (see material and methods). Consistent with endogenous protein, staining with antibodies specific for the pericentriolar material-1 (PCM1) revealed an intracellular pool of GFP-PRL-1 around the MTOC in non-stimulated cells (Figure 3A). To further characterize this pool, we stained the specimens for CD71, a marker of the recycling compartment, which has been shown to deliver the TCR to the IS (21). PRL-1 co-localized with CD71 positive endosomes, suggesting a mechanism for PRL-1 delivery to the IS (Figure 3A). Supporting this idea, GFP-PRL-1 was accumulated at the mature IS (Figure 3B). Distribution of PRL-1 was also studied in JK cells transfected with mCit-PRL-1 (see material and methods) (Supplementary Figure 2) and stained with antibodies directed against extracellular epitopes of CD3ε and LFA-1. PRL-1 clearly co-localized with LFA-1 areas. A partial co-localization was also found with CD3ε in more restricted sites through the IS (Figure 3C and Supplementary Figure 3).

### Dynamic delivery of PRL-1 to the immunological synapse

**Figure 4:**
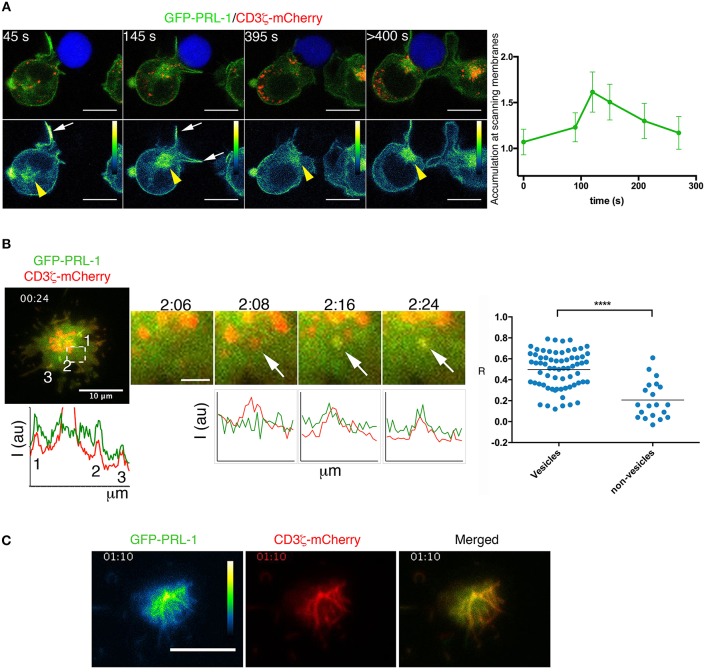
Dynamic delivery of PRL-1 to the IS. **(A)** Right panel: Frames of a representative time-lapse confocal microscopy experiment from 13 conjugates (*n* = 5 experiments) showing accumulation of GFP-PRL-1 at the IS. It is shown the distribution of GFP-PRL-1 in the pseudocolor image and in the merged image of channels (green signal). The red signal in merged images corresponds to CD3ζ-mCherry. The Raji cell is labeled with CMAC (blue). Time in seconds (s) is indicated. White arrows indicate transient accumulation of GFP-PRL1 in scanning membranes. Yellow arrowheads indicate the pericentriolar location of GFP-PRL-1. Scale bars 5 μm. The right graph represents the quantification of the accumulation of GFP-PRL-1 in scanning membranes along the time of complete (from the beginning) interactions. It is represented the mean and the SD obtained in 6 conjugates. **(B)** A merged image of the red (CD3ζ-mCherry) and the green (GFP-PRL-1) channel is shown of the representative time-lapse TIRFM experiment presented in Video 3. Lower and left graph represents the profile of the fluorescence intensity of the green and the red channel on the line that would cross the numbered sites. A magnified area of the region pointed by a square is shown at different times. White arrows point a CD3ζ-mCherry-containing vesicle in which GFP-PRL-1 arrives. Scale bar 2 μm. Intensity profiles show the correlation of the green and the red intensities at sites pointed by the arrows displayed in magnified areas. The right panel shows the Pearson's coefficient for the signal correlation of CD3ζ-mCherry and GFP-PRL-1 in vesicles arrived at the interface. Spots represent areas containing or not vesicles obtained from 5 cells (*n* = 3 experiments). Samples Were Compared by a T-test ^****^P < 0.0001. **(C)** Green (pseudocolor) and red channels, as well as, the merged image of a frame of the time lapse TIRFM experiment presented in Video 5. **(B,C)** A total of 11 cells were tracked in *n* = 3 experiments. Time in minutes:seconds is indicated.

The accumulation of PRL-1 at the IS regardless of the MTOC polarization (Figure 2B), suggested that PRL-1 was delivered to the contact site before the IS maturation. To further prove this idea the polarization of the GFP-PRL-1 fusion protein was also tracked in live cells from the initial scanning of the APC until the complete assembly of the IS. Time-lapse confocal microscopy revealed a fast and transient accumulation of GFP-PRL-1 at dynamic membranes scanning the APC and a subsequent delivery, along with the endosomal CD3ζ-mCherry, to the mature IS from the polarized pericentriolar compartment (Figure 4A and Supplementary Figure 4A, see white arrows and yellow arrowheads for membranes and intracellular compartment, respectively, and Supplementary Videos 1, 2). Membrane targeting and delivery of PRL-1 to the IS was dependent on the carboxyl terminal CAAX, as revealed by a mutant lacking this motif (Supplementary Figure 4B).

To further assess the co-localization of PRL-1 with CD3 sites at the established IS we used total internal reflexion microscopy (TIRFM), which enabled us to image cells with higher spatial and temporal resolution. Co-localization of GFP-PRL-1 and CD3ζ-mCherry was found at peripheral dynamic sites of the IS established by JK cells adhered to activating surfaces (see material and methods) (Figure 4B, Supplementary Figure 4C and Supplementary Videos 3,4). PRL-1 also located at CD3ζ-containing vesicles recruited to the interaction site (Figure 4B, see arrows in magnified areas). By tracking the early adhesion of JK cells in these assays, a clear localization of GFP-PRL-1 was observed in clusters enriched in CD3ζ-mCherry (Figure 4C and Supplementary Video 5). These data suggested that PRL-1 is included in CD3ζ-containing vesicles polarized to the IS and in signaling aggregates organized during initial T cell activation.

### Regulation of actin dynamics by PRL-1 catalytic activity

**Figure 5:**
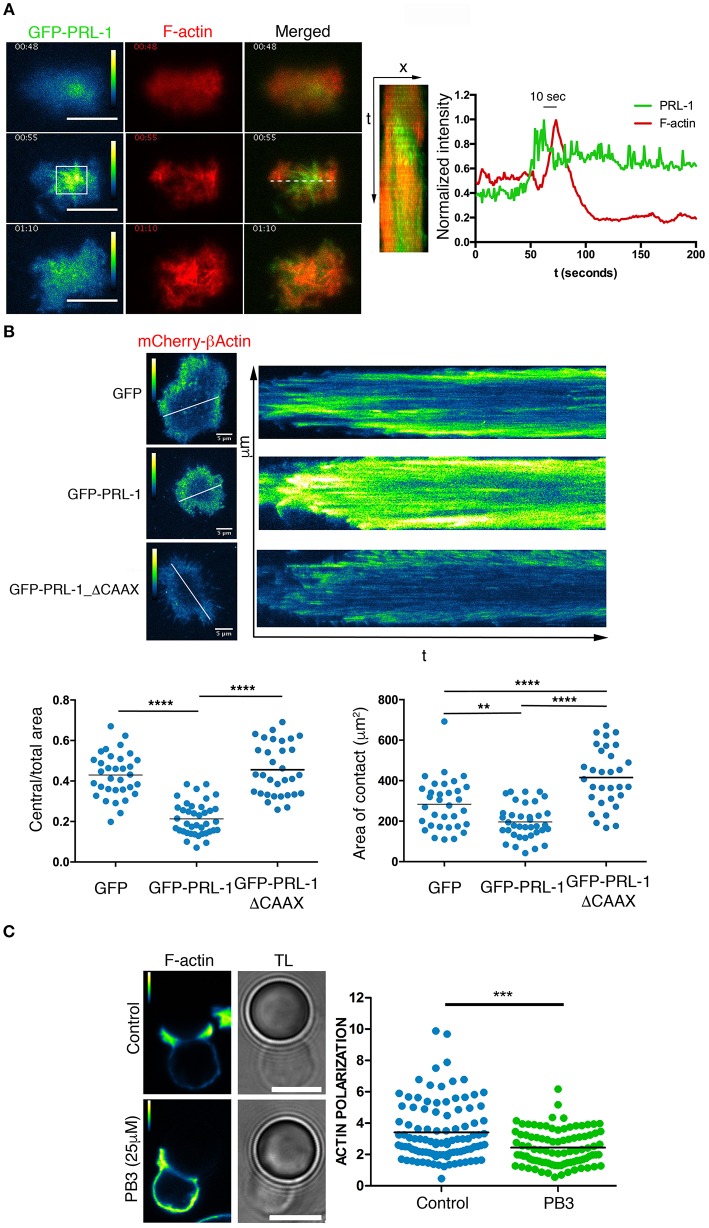
PRL-1 regulates actin dynamics at the IS. **(A)** Green (pseudocolor) and red channels and merged image of three frames of the time-lapse TIRFM experiment shown in Video 6. Scale bars 10 μm. Time in minutes:seconds is shown. A kymograph of the green and the red channel obtained from the line drawn in the merged image is shown. The normalized intensity profile is shown of the green and red channel in the square labeled in the pseudocolor image. The time in seconds (s) between the maximal intensity in both channels is indicated. A representative cell is shown of 5 cells tracked **(B)** Pictures show the mCherry-β-actin (pseudocolor) in frames and kymograph of representative time-lapse TIRFM experiments of cells also expressing GFP-PRL-1, GFP-PRL-1-ΔCAAX, or GFP alone. Kymographs were obtained in lines shown in the frame. Calibration bar is indicated. Dots in lower graphs represent individual cells adhered to the activating surface obtained from *n* = 6 experiments. Different samples were compared by the one-way ANOVA. Asterisks represent the *p*-value: ^**^P ≤ 0.01, ^****^P ≤ 0.0001 **(C)** Immunofluorescence of IS-like structures stained for F-actin. The green (pseudocolor) and the transmission light (TL) are shown. The PB3 treatment (25 μM) and the control (vehicle) are indicated. Scale bars 5 μm. Quantification of the F-actin accumulation at the interface is shown in the right panel. Dots in the graph represent individual cell/bead conjugates analyzed in n = 4 experiments. Samples were compared by a *t*-test. Asterisks represent the *p*-value: ^***^P ≤ 0.001.

The recruitment of PRL-1 at the IS during the early activation times suggested a regulatory role of this enzyme in early events leading to the IS assembly. Thus, this possibility was further investigated by TIRFM of IS-like structures as before. Initially, we tracked the formation of F-actin by using the LifeAct probe. An early transient enrichment of GFP-PRL-1 preceded actin polymerization. Maximal enrichment of PRL-1 accumulation at the adhesion site occurred 10 s before the maximal amount of F-actin (Figure 5A and Supplementary Video 6). These observations suggested a regulatory role of PRL-1 in the actin polymerization, which mediates early adhesion to the activating surface. To test this possibility JK cells were co-transfected with a plasmid expressing mCherry-β-actin under a speckle promoter and plasmids expressing GFP-PRL-1, GFP-PRL-1_ΔCAAX, or GFP alone, used as control. This model enabled us to monitor the formation of the distal (d)SMAC/lamelipodium containing the retrograde flow of actin and surrounding a wide central area cleared of actin (4, 22). While cells overexpressing GFP or GFP-PRL-1_Δ_CAAX showed a typical formation of a narrow dSMAC, cells overexpressing GFP-PRL-1 showed a smaller central area cleared of actin (Figure 5B). GFP-PRL-1 transfected cells also showed a reduced area of contact in comparison with cells expressing GFP and cells expressing GFP-PRL-1-ΔCAAX, which developed the largest contact area (Figure 5B). Consistent with previous data, experiments showed the delivery of PRL-1 to the IS in two waves; an initial transient delivery to actin enriched scanning membranes and a later delivery to the central area of the IS once dynamic actin reached the periphery (Supplementary Figure 5 and Supplementary Video 7).

We further studied whether the catalytic activity of PRL-1 regulated F-actin dynamics during T cell activation by using procyanidin B3 (PB3), a selective inhibitor of PRL-1 catalytic activity (23). Peripheral blood CD4 T cells were enabled to form IS-like structures with micro-spheres coated with anti-CD3ε and anti-CD28 antibodies, and F-actin polymerization at the IS was evaluated by phalloidin staining. Polymerization of actin at the IS was significantly reduced in cells treated with the inhibitor when compared with cells exposed to the vehicle as control (Figure 5C).

Taken together, these data suggest that the catalytic activity of PRL-1 regulates actin dynamics during the assembly of the IS.

### Enzymatic activity of PRLs is required for optimal IL-2 secretion

**Figure 6:**
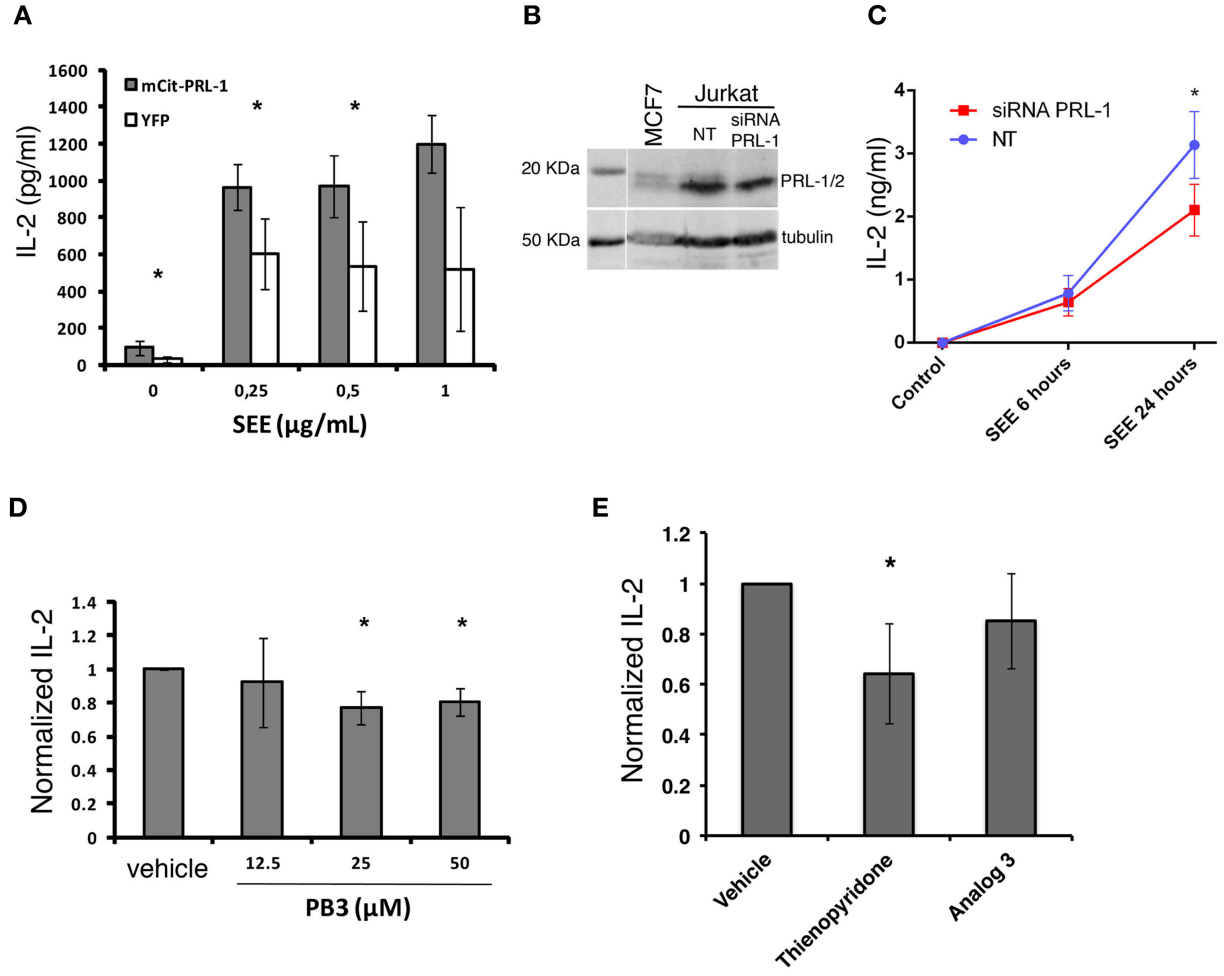
PRL-1 regulates IL-2 secretion. **(A)** IL-2 secretion assessed by ELISA in JK cells overexpressing either mCit-PRL-1 or YFP alone and stimulated for 16 h with Raji cells loaded with SEE. Samples in each SEE concentration were compared by a paired *t*-tests (*n* = 3 experiments). **(B)** PRL-1 and PRL-2 protein expression in JK cells transfected with a pool of siRNAs targeting PRL-1 or with a pool of non-targeting siRNAs (NT) as control. A lysate of the breast cancer derived cell line MCF7 was included in the WB experiment represented. **(C)** IL-2 secretion assessed by ELISA in JK cells transfected with the PRL-1 siRNA pool or the NT. The mean ± the SEM of *n* = 3 experiments is shown. Samples at different time points were compared by a *t*-test. The asterisks represent the *p*-value: ^*^P ≤ 0.05. **(D)** IL-2 secretion assessed by ELISA in peripheral blood CD4 T cells treated or not with PB3 or the vehicle and stimulated for 6 h with antiCD3ε/CD28-coated beads. Concentrations of PB3 are indicated. Results were normalized to control (Vehicle) and the mean ± SD is shown of the data obtained with *n* = 4 experiments. Samples in each PB3 concentration were compared with the control (Vehicle) by a one-sample *t*-test of one tail. Asterisks represent the *p*-value: ^*^P ≤ 0.05 **(E)** IL-2 secretion assessed by ELISA in peripheral blood CD4 T cells treated or not with the indicated inhibitor or the vehicle and stimulated for 6 h with anti-CD3ε/anti-CD28 coated beads. Results were normalized to control (Vehicle) and the mean ± SD is shown of the data obtained with *n* = 4 experiments. Samples were compared with the control (vehicle) by a one-sample *t*-test. Asterisks represent the *p*-value: ^*^P ≤ 0.05.

The delivery of PRL-1 to the IS and fostering of acting dynamics prompted us to study whether PRL-1 regulated the activation or the effector function of CD4 T cells. Secretion of IL-2 was analyzed in JK transfected with the plasmid coding for mCit-PRL-1 fusion protein and cultured for 16 h with Raji B cells loaded or not with SEE. An enhanced secretion of IL-2 was observed in cells overexpressing the mCit-PRL-1 fusion protein compared with those cells expressing the YFP alone (Figure 6A and Supplementary Figure 6). Interfering the endogenous expression of PRL-1 by small interference (si)RNA also decreased the amount of IL-2 detected in supernatants of JK/Raji(SEE) conjugates (Figures 6B,C). In order to delimit the step in which PRL-1 participates in the IL-2 response, JK cells were also stimulated with PMA and ionomycin. Also in this case, treatment of cells with siRNA specific for PRL-1 decreased the detection of IL-2 in supernatants (Supplementary Figure 7).

In order to assess the requirement of the catalytic activity of PRL-1 in this process, pharmacological inhibition of its catalytic activity was done in peripheral blood CD4 T cells by using PB3. Consistent with previous experiments, PB3 caused a subtle decrease in the amount of IL-2 secreted by peripheral blood CD4 T cells stimulated for 6 h with anti-CD3ε and anti-CD28 antibodies (Figure 6D). Given the high identity of the three PRLs, we also used Thyenopiridone (TP), a selective inhibitor of the enzymatic activity of the group of PRLs (24) (Figure 6E). TP treatment of human CD4 T cells stimulated with anti-CD3ε and anti-CD28 antibodies also decreased the secretion of IL-2. However, no inhibitory effect was found when cells were pre-treated with Analog 3, a drug that also inhibits classical PTPs (25), such as the negative regulator of TCR signaling TC-PTP (26). Thus, these data suggest that the enzymatic activity of the group of PRLs was required for the optimal secretion of IL-2.

## Discussion

In this study, we have analyzed the expression of the PRLs in human peripheral blood CD4 T cells, the dynamic delivery of PRL-1 at the IS and the role of this protein during IS assembly and T cell activation. PRL-1 is early delivered to the IS in early scanning membranes and later in pericentriolar vesicles. PRL-1 accumulation at adhesion sites, in which actin polymerizes, suggests a causal relation between both events. Indeed, pharmacological inhibition indicates that the catalytic activity of PRL-1 participates in actin rearrangements during IS assembly. Polarization of PRL-1 in two different pools suggests the existence of several roles of this enzyme during T cell activation and/or effector function. In this regard, PRL-1 catalytic activity seems to contribute to these processes as determined by IL-2 secretion.

Previous data on the expression of *PTP4A1* and *PTP4A2* in lymph nodes (17) suggested a regulatory role of PRLs in the adaptive immune responses by mature lymphocytes. Our results support this notion; we show a high expression of the genes coding for PRLs in human peripheral blood CD4 T cells and a contribution of their phosphatase activity to the TCR-induced secretion of IL-2. Although PRL-2 expression levels are higher than those of PRL-1, T cell activation by phorbol esters and Ionomycin downmodulates PRL-2 expression and, consequently, raises the relative amount of PRL-1 regarding PRL-2. The balanced expression of PRL-1 and PRL-2 induced by activation suggested a predominant role of PRL-1 during tissue inflammation. This idea and the regulation of the actin cytoskeleton by the catalytic activity of PRL-1 (18), prompted us to study the dynamics and the regulatory role of PRL-1 during T cell activation, a process dependent on actin rearrangements.

Live-cell confocal and TIRF microscopy data suggest that intracellular PRL-1 is delivered to the IS in two stages: early at F-actin- and CD3ζ-enriched sites and, later, from the polarized intracellular compartment organized around the MTOC. The co-localization of pericentriolar PRL-1 with CD71 and the location in CD3ζ-containing vesicles suggests that PRL-1 traffics to the IS in the TCR-containing recycling endosomes described previously (21). Once at the established IS, PRL-1 mainly locates at LFA-1 and, to a lower extent, at CD3 sites. The endosomal compartment has been shown to be essential for T cell activation and cytokine secretion (2, 27–29). It will be of interest to further characterize the particular endosomes that convey PRL-1 to the IS and to analyze whether the endosomal traffic and/or the signal from the TCR or LFA-1 is regulated by this molecule.

Early localization of PRL-1 at CD3ζ and β-actin sites immediately follows the adhesion of Jurkat cells to activating surfaces. This is reminiscent of signaling aggregates previously shown (22) and suggests a regulatory role of PRL-1 in cytoskeleton rearrangements downstream TCR/CD28 signaling. Overexpression and pharmacological inhibition of the catalytic activity support the notion that PRL-1 is required for proper actin dynamics and cell spreading. The data obtained with the GFP-PRL-1-ΔCAAX mutant suggest that membrane targeting is required for actin regulation by this phosphatase. Consistent with previous reports (18), our data indicate that the regulation of the actin dynamics involves the catalytic activity and, consequently, the existence of PRL-1 substrates at the early IS. In this cell context, PRL-1 might regulate the signaling pathway triggered by the TCR/CD28 or LFA-1 or might constitute a downstream cytoskeleton regulator. Consistent with the later idea, different proposed substrates for the homolog PRL-3, including Ezrin and PIP_2_ (16), are important elements of cytoskeleton regulation (21).

The actin dynamics is essential for intracellular signaling and IL-2 production upon TCR engagement (30, 31). Accordingly, we have found that the catalytic activity of PRL-1 contributes to IL-2 secretion. It should be nonetheless noted that the data presented here indicate that PRL-1 regulates IL-2 response by acting on steps downstream PKC activation and Ca^2+^ raise. Importantly, components of the cellular machinery such as the cytoskeleton and the endosomal-recycling compartment, both crucial for proper sustained activation and IL-2 production, are regulated downstream PKC and Ca^2+^ (4, 29, 32–35). Complex feedback loops, which we don't completely understand yet, are apparently occurring (36, 37). Thus, the cellular machinery builds a three-dimensional IS, which is essential for proper sustained signaling, transcription factor activation and effector function. PRL-1 seems to participate in this IS architecture.

The stronger inhibitory effect on IL-2 secretion by pharmacological inhibition of PRLs than of PRL-1 indicates a redundant contribution of PRL-2 and/or PRL-3 in this effector function. This is consistent with the high identity of the primary structure of the three human PRLs, 87% (PRL-1 vs. PRL-2), 79% (PRL-1 vs. PRL-3) and 76% (PRL-3 vs. PRL-2) (16). Supporting a redundant role during T cell activation, the three molecules have been found polarized to the IS (Castro-Sánchez and Roda-Navarro, unpublished data). Polarized secretion of IL-2 to the IS has been described (38). It is tempting to speculate that PRLs might be involved in the polarized secretion of cytokines. We envisage that regulatory mechanisms of PRLs during IS assembly might contribute to T cell polarity during T cell effector functions.

The fast and transient delivery of PRL-1 at membranes scanning the APC makes conceivable that a cytosolic pool of PRL-1 represent a fast diffusible signaling intermediate able to reach different intracellular signaling-competent compartments. PRL-1 is a cytosolic protein containing a polybasic sequence and a C-terminal CCAX motif farnesylated in the first cysteine (Cys170). We have observed that this motif is essential for targeting the protein at membranes and at the IS. *In vitro* experiments have previously shown the palmitoylation of the homolog PRL-3 in the second cysteine of the motif (Cys171) (39), suggesting that this posttranslational modification might have a role in the dynamics of the PRLs. Studying mobility properties of PRL-1 and the regulatory role of posttranslational modifications will assist in our understanding of its delivery and function at the IS.

The activation of T cells in the lymph node involves serial and transient interactions followed by later stable interactions of T cells with dendritic cells (DC) (40). Our data would be consistent with a regulatory role of PRL-1 in transient and/or stable cognate naïve T cell-DC interactions or during T-B cell cooperation. Thus, there is a new open research avenue focus on the function of PRLs during immune responses by lymphocytes.

## Materials and methods

### Cell purification and culture

Peripheral blood mononuclear cells (PBMCs) were obtained by Lymphoprep^TM^ (Rafer, Spain) density gradient centrifugation. CD4^+^ T cells were isolated from PBMCs by negative selection with Dynabeads^TM^ Untouched^TM^ Human CD4 T cells kit (Invitrogen CA, USA). CD4^+^ naïve T cells were isolated from PBMCs by negative selection with Naïve CD4^+^ T Cell Isolation Kit II (Miltenyi Biotec, Germany). Purity 90–95% of CD3^+^/CD4^+^/CD45RA^+^ cells was assessed by flow cytometry (FACS). Purified cells were polarized to Th1 effector cells by activation with Dynabeads Human T-Activator CD3/CD28 (Invitrogen) in RPMI 1640 culture medium (Lonza Group, Switzerland) supplemented with 10% FCS (Gibco, MD, USA), 2 mM L-Glutamine, 100 U/mL penicillin, 100 μg/ml streptomycin (all from Lonza Group) and 10 ng/mL of IL-12 (Peprotech, NJ, USA). *In vitro* polarization to Th1 was assessed by intracellular staining for IFN-γ after 4 h stimulation with 10 ng/mL Phorbol 12-myristate 13-acetate (PMA) and 1 μM Ionomycin (both from Sigma Aldrich, MO, USA) (data not shown). The Raji and Hom2 B cell lines, used as APCs, and CD4 T cell line Jurkat (JK) clones J77 (41) and CH7C17 (here called CH7) (42) were grown in the same medium as primary cells, but without IL-12. The CH7 stably express Vβ3 TCRs specific for influenza Hemagglutinin (HA) peptide and its culture medium was supplemented with 4 μg/ml puromycin and 0.4 mg/ml hygromycin B to keep expression of transfected HA-specific Vβ3 TCRs. FACS data were acquired with a FACSCalibur system (BD) and analyzed by the Flowjo software (Treestar, Inc., OR, USA). Primary cell isolation in the project was approved by the Clinical Research Ethical Committee of the San Carlos Clinical Hospital.

### Antibodies and reagents

Mouse fluorescently labeled antibodies specific for CD4 and CD45RA were obtained from Beckman Coulter (CA, USA), APC-labeled anti-CD3 from BD Pharmingen (CA, USA), rabbit anti-GFP from Life Technologies (CA, USA) and mouse anti-α tubulin and mouse IgG1 isotype control (MOPC21) from Sigma Aldrich (MO, USA). Anti-CD3ε (T3b) and anti-LFA-1 (TP1/40) supernatants were provided by Dr. Francisco Sanchez-Madrid (Hospital Universitario de la Princesa, Madrid, Spain). The mouse anti-PRL-1 ascites was provided by Dr. Qi Zeng (Institute of Molecular and Cell Biology, Singapore) and the rabbit anti-CD3ζ (CD247) by Dr. Balbino Alarcón (Centro de Biología Molecular Severo Ochoa, CSIC, Madrid, Spain). The anti-CD3, clone UCHT1 was purified from the hybridome (43) and the anti-CD28 obtained from BD Biosciences. Rabbit anti PCM-1 was obtained from Cell Signaling (MA, USA), mouse anti-PRL-1/2 (clone 42) from Millipore (MA, USA). Phalloidin-488, secondary antibodies goat anti-mouse-Ig Alexa 594, donkey anti-rabbit Alexa 488, goat anti-mouse Alexa 488 (all of them highly cross-absorbed) and the fluorescent tracker chloromethyl derivative of aminocoumarin (CMAC) were obtained from Molecular probes (OR, USA). Poly-L-lysine was obtained from Sigma Aldrich, mouse serum from Calbiochem (Germany) and Staphylococcus Enterotoxin E (SEE) from Toxin Technologies (FL, USA). PRLs inhibitors Analog 3 and thienopyridone were obtained from Enamine (Ucrania) and the PRL-1 inhibitor procyanidin B3 (PB3) from ChemFaces (Wuhan, PRC). ICAM1-Fc was produced as previously described (44).

### Expression plasmids, siRNA and cell transfection

PRL-1 and PRL-1_ΔCAAX cDNAs were amplified by RT-PCR and cloned at the XhoI and BamHI restriction sites of the pEGFP-C1 vector from Clontech Laboratories (CA, USA). The resulting constructs encode the GFP-PRL-1 and GFP-PRL-1-ΔCAAX. mCitrine-PRL-1 (mCit-PRL-1) and mCitrine-PRL-2 (mCit-PRL-2) encoding constructs were kindly provided by Dr. Philippe Bastiaens (Max Planck Institute of Molecular Physiology, Dortmund, Germany). The expression vector coding for the CD3ζ-mCherry chimeric protein was kindly provided by Dr. Balbino Alarcón. For actin visualization in living cells we used the expression vector pCAGLifeAct–TagRFP from Ibidi (Germany) or the Speckle mCherry-β-actin plasmid kindly provided by Dr. M. Vicente-Manzanares (Centro de Investigación del Cancer, Salamanca, Spain). Two million JK cells were nucleofected with 4 μg of the indicated plasmid. 24 h after transfection, living cells were isolated by centrifugation on Lymphoprep^TM^ and used for experiments.

For siRNA experiments, we nucleofected 300 nM/million cells of a pool of three PTP4A1 siRNAs [ON-TARGETplus J-006333-06 (GAUUGUUGAUGACUGGUUA), J-006333-07 (CCAAUGCGACCUUAAACAA) and J-006333-09 (GAAAGAAGGUAUCCAUGUU)] (Dharmacon, Inc). Experiments were done 48 h after nucleofection. As control, we used the siRNA ON-TARGETplus Non-targeting (NT) Pool (Dharmacon, Inc).

Nucleofection was done with the Amaxa® Nucleofector® II device using Amaxa® Cell Line Nucleofector® Kit V (both from Lonza Group) by using program X-001.

### Conjugation assays

JK cells were conjugated with Raji cells loaded with SEE (1 μg/mL) or Hom2 loaded with HA peptide (100 μg/ml). Raji and Hom2 were identified on cell conjugates by labeling with 10 μM CMAC. For cell conjugation assays JK were mixed with the APCs at a cell ratio 1:1, briefly centrifuged, and gently resuspended before being plated on poly-L-Lysine-coated coverslips. Cells were then allowed to interact for around 20 min. Human primary CD4 T cells were conjugated with anti-CD3/anti-CD28 coated microspheres (Sigma Aldrich) at 1:1 ratio for 20 min. When specified, cells were pre-incubated for 1 h with PB3, which was maintained during the whole stimulation time. Both JK/Raji and CD4/microspheres conjugates were then fixed with 4% paraformaldehyde in PBS during 5 min at room temperature. After fixation, conjugates were stained with the indicated antibodies. For live-cell time-lapse confocal microscopy, JK cells co-transfected with GFP-PRL-1 and CD3ζ-mCherry were attached to the bottom of LabTek chambered cover glasses (Nunc, Denmark) coated with 20 μg/ml poly-L-Lysine. Then, Raji cells labeled with CMAC were added. Cells were imaged in DMEM without phenol red (Lonza) supplemented with 5% FCS and 25 mM hepes buffer (Gibco) at 37°C and 5% of CO_2_.

### Fluorescence microscopy

Confocal microscopy was performed with a FV-1200 microscope (Olympus Deutschland GmbH, Germany). 405 nm (for the CMAC), 488 nm (for the GFP, Alexa488 and mCitrine) and 594 nm (for the Alexa594 and mCherry) excitation lines were used. The elapsed time is indicated in each time-lapse microscopy experiment.

For TIRFM, cells were diluted in imaging medium (Hank's balanced salt solution (HBSS) supplemented with 1% fetal bovine serum and 25 mM HEPES). Cells were allowed to settle onto glass bottom dishes (No 1.5 Mattek; Ashland, MA, US) coated with ICAM1-Fc (10 μg/ml), anti-CD3ε (10 μg/mL) and anti-CD28 (3 μg/ml). Cells were immediately visualized with a Leica AM TIRF MC M system mounted on a Leica DMI 6000B microscope coupled to an Andor-DU8285_VP-4094 camera and fitted with a HCX PL APO 100.0x1.46 OIL objective. Images were processed with the accompanying confocal software (LCS; Leica, Germany). The laser penetrance used was 150 or 200 nm for both laser channels (488 and 561 nm), using the same objective angle. Optimized time-lapse settings are specified throughout the text. Synchronization was performed with the accompanying Leica software.

All processing, analysis and quantification of fluorescence images were developed with FIJI freeware (NIH, USA). Polarization of molecules to the synapse or at membranes scanning APCs was quantified using SynapseMeasures Plugin as described (45). Briefly, after background subtraction, fluorescence intensity in regions placed at the IS surface interface was normalized to fluorescence intensity in regions placed at the cortical cytoplasm and non-IS membranes of the cell. Co-localization was quantified by the Pearson's coefficient.

### IL-2 secretion

Mixtures of JK cells (transfected with either mCit-PRL-1 or YFP) and Raji cells (loaded or not with SEE) or primary CD4 T cells and Dynabeads™ Human T-Activator CD3/CD28 (Gibco) were done at cell ratio of 1:1 during overnight or 6 h cultures, respectively. In addition, cells were also stimulated for 6 h with 10 ng/mL PMA and 1 μM Ionomycin. Supernatants were then collected for IL-2 measurements. When specified, cells were pre-incubated for 1 h with the indicated inhibitor, which was maintained during the whole stimulation time, or transfected with the indicated siRNA. The IL-2 content in supernatants was determined by ELISA assays using the BD OptEIA^TM^ ELISA set (BD). Quantification was done in an ELx800 absorbance microplate reader (Biotek, VT, USA).

### Western blot

Transfected cells were lysed for 30 min in ice-cold RIPA buffer (20 mM Tris-HCl pH 7,5; 1% NP-40; 0,5% sodium deoxycholate; 0,1% SDS; 150 mM NaCl; 10 mM β-glicerophosphate; 1X protease inhibitor cocktail; 10 mM NaF; 1 mM PMSF; 1 mM Na_3_VO_4_) and sonicated in a digital sonifier (Branson). Cell lysates were mixed with 4x Laemmli buffer + 20% β-mercaptoethanol. Samples were then boiled at 95°C for 5 min and proteins were separated by SDS-PAGE in acrylamide gels which were then transferred to an Immobilon-FL membrane (Millipore). After transference, the membranes were blocked with LICOR blocking buffer before O/N incubation with primary mouse or rabbit antibodies. HRP-conjugated goat anti-mouse antibody and Pierce^TM^ ECL Plus Substrate or fluorescently-labeled secondary antibodies IRDye 680 goat anti-Mouse and IRDye 800 goat anti-rabbit were used. All blots were scanned and fluorescence or quimioluminiscence was quantified with an Odyssey Infrared Imager (LICOR).

### Quantitative PCR

CD4 or Th1 cells were stimulated for 10, 30, 60, 120, and 240 min with 10 ng/mL PMA plus 1 μM Ionomycin. RNA was isolated by the Absolutely RNA Microprep Kit (Agilent Technologies). qPCR was performed with Taqman assays of Applied Biosystems (CA, USA). Taqman probes used were Hs00743856_s1 (PTP4A1), Hs00754750_s1 (PTP4A2), Hs02341135_m1 (PTP4A3), Hs00959886_g1 (*PTPN2*), Hs00169359_m1 (*PTPN6*), Hs00978680_m1 (*PTPN7*), Hs04189704_m1 (*PTPRC*), Hs00934033_m1 (CD69), and Hs00272002_m1 (*GNB2L1*, housekeeping gene). Reactions were run in 7900HT Fast Real-Time PCR system (Applied Biosystems). Comparative analysis of the results obtained in different samples was done by using the delta Ct (ΔCt; Ct of the studied gene subtracted by the Ct of the housekeeping gene) or by the method 2^−ΔΔ*Ct*^ (46) as indicated.

### Statistical analysis

Statistical analysis was implemented with PRISM 6 (GraphPad, CA, USA) and Statgraphics Centurion XVII (Statpoint Technologies, Inc. VA, USA). When comparing three or more samples, one-way ANOVA followed by Bonferroni's multiple comparing test correction was used. When comparing two samples, Student *T*-test was performed. Welch correction was applied when variances between samples were statistically different. Normalized values were analyzed by a one-sample *T*-test. All tests were implemented with two tails with the exception of the indicated experiments.

## Author contributions

PC-S did the experiments, analyzed data, and contributed to the manuscript writing. RR-M, OA-S, SA-G, and SH-P contributed to the experiments. RR, CC, and QZ contributed important reagents. NM-C and FS-M participated in TIRFM experimentation and data analysis and corrected the manuscript. PR-N conceived and designed the research, analyzed data, and wrote the final manuscript.

### Conflict of interest statement

The authors declare that the research was conducted in the absence of any commercial or financial relationships that could be construed as a potential conflict of interest.
