# Supplementary material for: Phosphatase of Regenerating Liver-1 (PRL-1) Regulates Actin Dynamics During Immunological Synapse Assembly and T Cell Effector Function
Source: Front Immunol. 2018 Nov 20;9:2655. doi: 10.3389/fimmu.2018.02655 (PMC6255827; doi:10.3389/fimmu.2018.02655)
Supplement: Supplementary file 8 [file Data_Sheet_1.docx]

Supplementary Material

**Phosphatase of regenerating liver-1 (PRL-1) regulates actin dynamics during Immunological Synapse assembly and T cell effector function**

**Patricia Castro-Sánchez^1^, Rocío Ramirez-Munoz^1^, Noa B. Martín-Cófreces^2^, Oscar Aguilar-Sopeña^1^, Sergio Alegre-Gomez^1^, Sara Hernández-Pérez^1^, Raquel Reyes^3^, Qi Zeng^4^, Carlos Cabañas^1,3^, Francisco Sánchez-Madrid^2^, and Pedro Roda-Navarro^1^***

*** Correspondence:** Pedro Roda-Navarro: [proda@med.ucm.es](mailto:proda@med.ucm.es)

# Supplementary Figures and movie legends

## Supplementary Figures

**
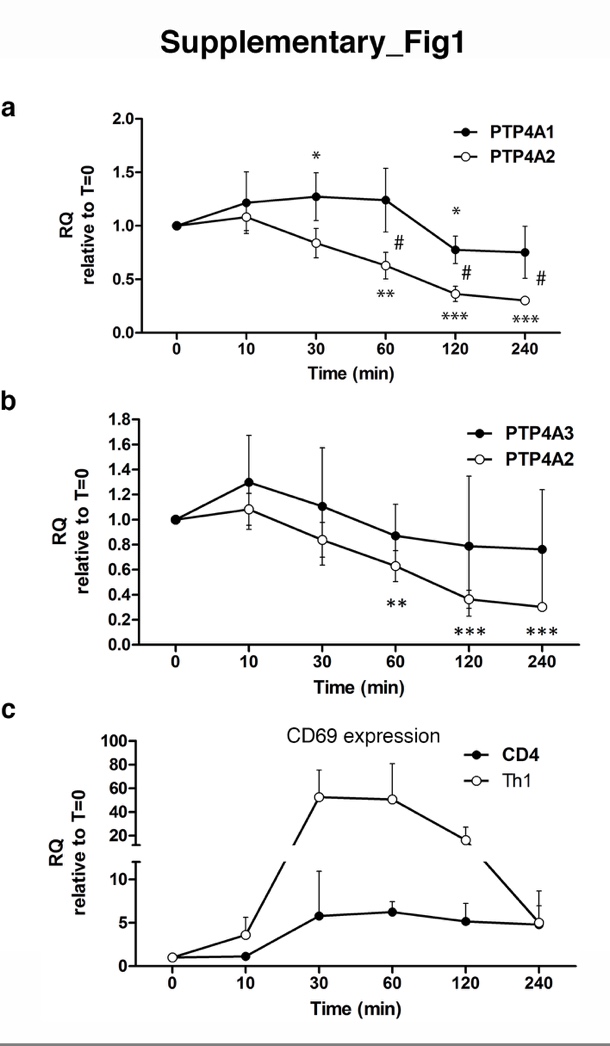
**

**Supplementary Figure 1: Expression of *PTP4A1*, *PTP4A2* and *PTP4A3*** **(a)** Expression of *PTP4A1* and *PTP4A2* mRNA in *ex vivo* isolated peripheral blood CD4 T cells upon stimulation with PMA and Ionomycin for the indicated times in minutes (min). Graphs represent the relative expression (RQ) with respect to t=0. The mean ± SD is shown of RQ values obtained from n=4 different donors. Asterisks indicate the p-value of a one-sample t-test comparing each time to t=0. Hashes indicate the p-value of a t-test comparing *PTP4A1* and *PTP4A2* expression at each time. * and # P≤0.05, ** P≤0.01, *** P≤0.001. **(b)** Expression of *PTP4A3* compared to the same data of *PTP4A2* presented in A. Graphs represent the mean ± SD of the relative expression (RQ) with respect to t=0 in the same samples and donors analyzed in A. Asterisks indicate the p-value of a one-sample t-test comparing each time to t=0. ** P≤0.01, *** P≤0.01. **(c)** Induction of CD69 mRNA in the same samples and donors analyzed in A or in figure 1C of the main text. Graphs represent the mean ± SD of the relative expression (RQ) with respect to t=0.


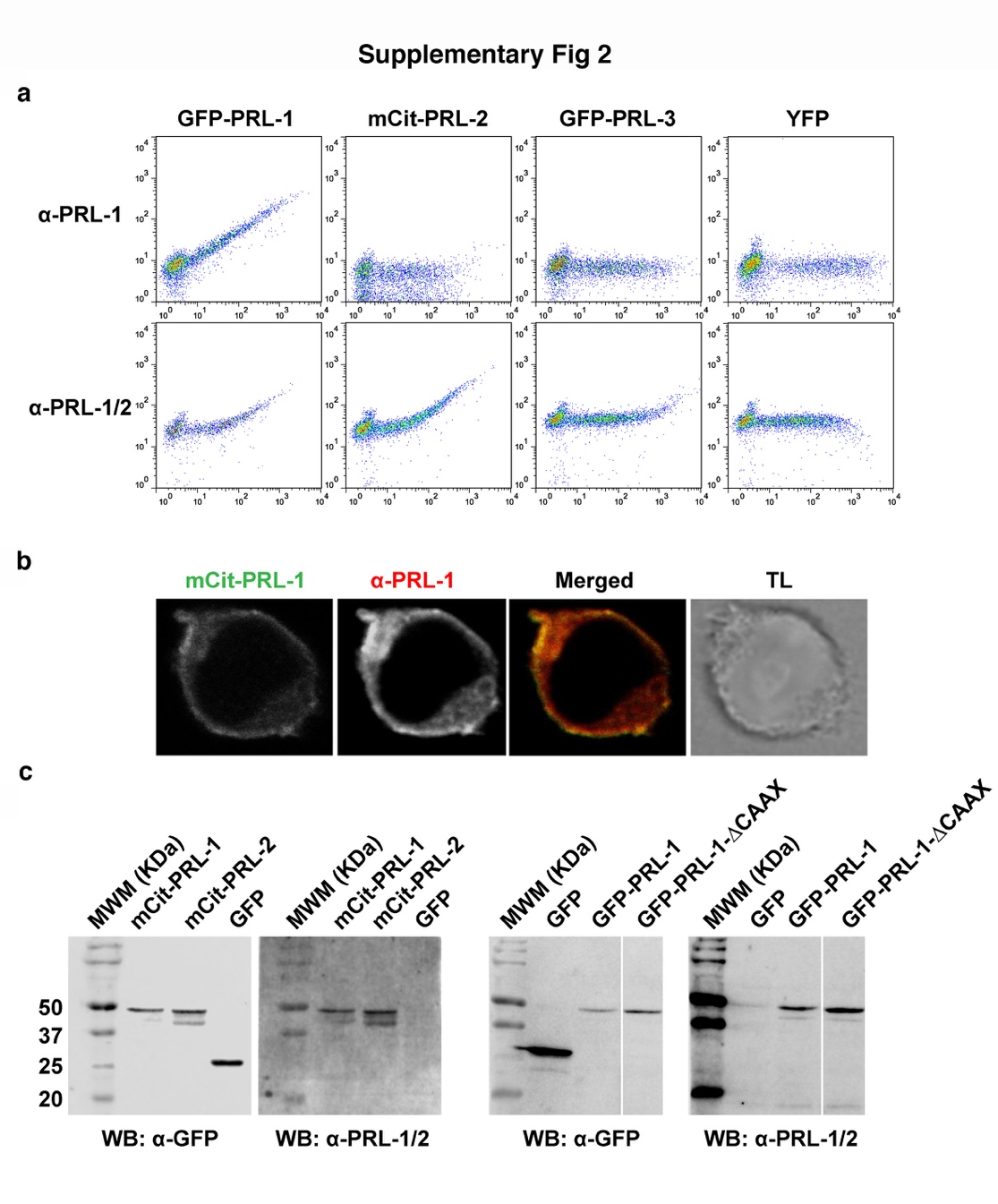


**Supplementary Figure 2. Analysis of the integrity of PRL-1 fluorescent proteins and antibody specificity.** (**a**) JK cells overexpressing GFP-PRL-1 and mCit-PRL-2, as well as GFP and YFP alone were stained with two different mouse anti-PRL-1 antibodies (material and Methods) and analyzed by flow cytometry. The x (FL1) and y (FL4) axes correspond to the fluorescence intensity of the GFP and the antibody staining, respectively. (**b**) Images are shown of the green and red channels (grey scale), as well as the merged and transmission light (TL) images of a confocal section of a representative JK cell transfected with mCit-PRL-1 and stained with the specific anti-PRL-1 antibody. (**c**) Western blot for PRL-1 and PRL-2 detection in JK cells transfected with the indicated plasmids. The antibodies used are described in material and methods. (**a**-**c**) Representative data are shown.

**
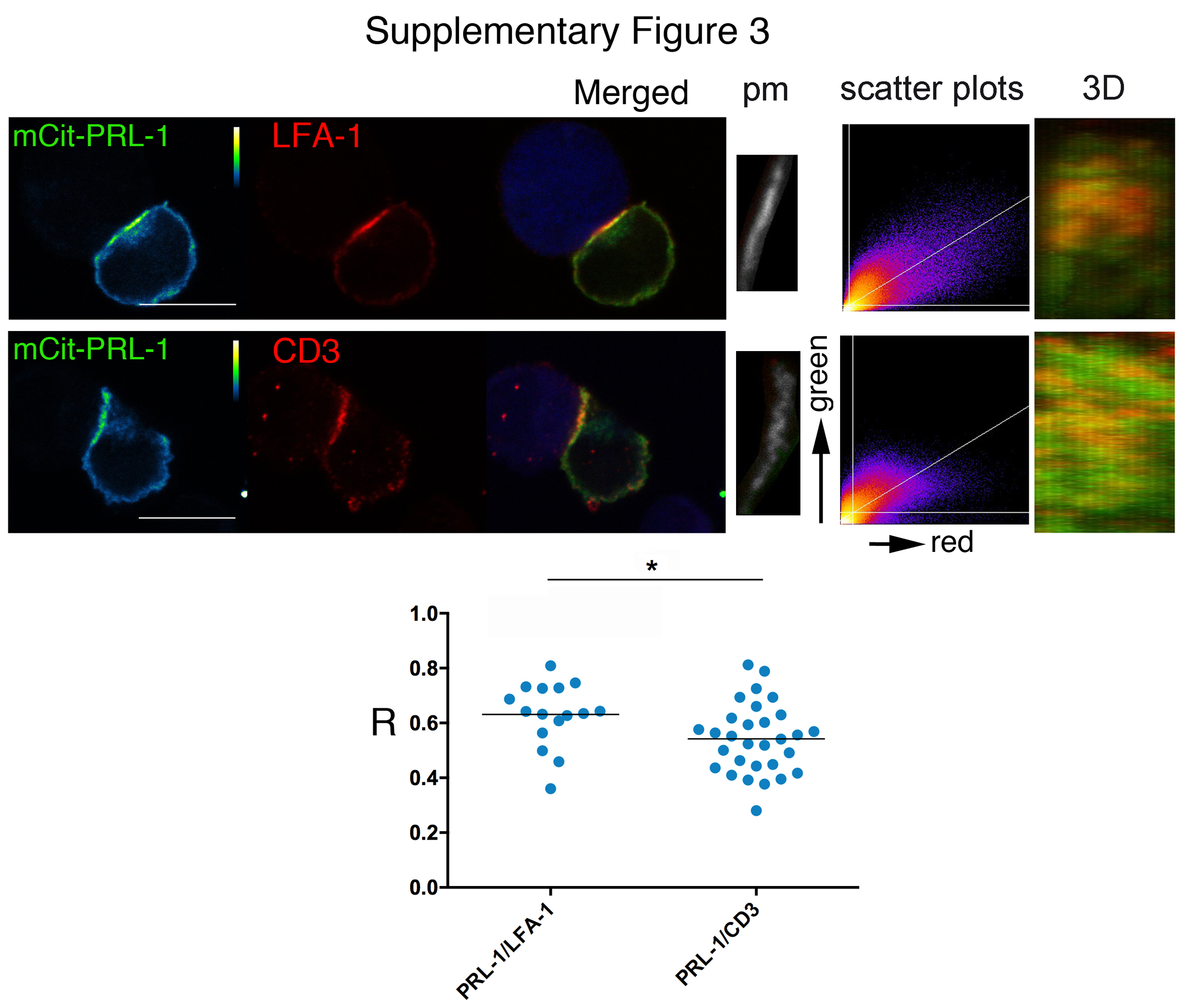
**

**Supplementary Figure 3. Subcellular distribution of PRL-1 at the IS organized in JK cells**. PRL-1 Immunofluorescence of cell conjugates formed by JK (CH7C17) cells overexpressing mCit-PRL-1 and conjugated with Hom2 cells labeled with CMAC (blue) and loaded with influenza Hemaglutinin (HA) peptide. The IS markers are shown in red. mCit-PRL-1 is shown as a pseudocolor image with the calibration bar. Co-localization is shown in a pixel map (pm) obtained at the interaction site. White pixels indicate co-localization sites. Scatter plots of green and red channels along the stack are shown. The interface surface obtained from a 3D reconstruction of the IS where co-localization was analyzed is shown. Scale bars 10 μm. Lower graph: Quantification of the co-localization by Pearson coefficients (R). Dots represent individual cell conjugates obtained from n=4 (PRL-1 vs LFA-1) and n=8 (PRL-1 vs CD3) expereiments. Samples were compared by a t-test. Asterisks represent the p-values: *P≤0.05.

**
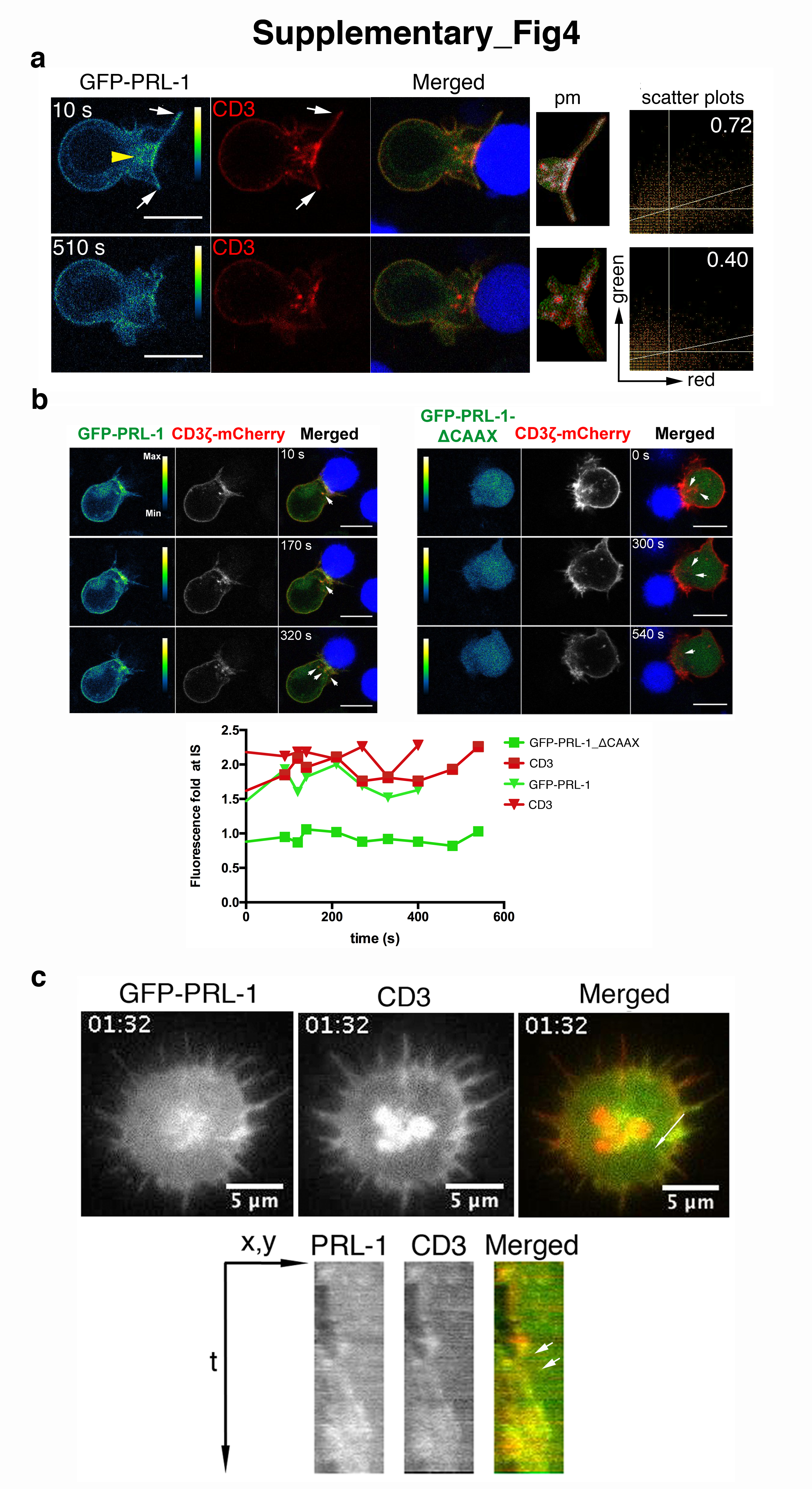
**

**Supplementary Figure 4. Dynamic distribution of GFP-PRL-1 at the established IS. (a)** An early and a late frame are shown of the representative time-lapse confocal microscopy experiment presented in Movie 2. It is shown the distribution of GFP-PRL-1 in pseudocolor and merged images (green signal), as well as CD3ζ-mCherry in red and merged images (red signal). The SEE-loaded RAJI cell is labeled with CMAC (blue). Time in seconds (s) is indicated. Co-localization is shown in a pixel map (pm) obtained at the interaction site. White pixels indicate co-localization sites. Scatter plots of green and red channels are shown. Numbers indicate Mander’s coefficients (MC). White arrows indicate the localization of GFP-PRL1 and CD3ζ-mCherry in scanning membranes. Yellow arrowhead indicates the intracellular compartment containing GFP-PRL-1 and CD3ζ-mCherry. Scale bar 5 μm. **(b)** Frames are shown of a representative time-lapse confocal microscopy experiment of JK cells expressing CD3ζ-mCherry and either GFP-PRL-1 or GFP-PRL-1_ΔCAAX and interacting with SEE-loaded Raji cells loaded with CMAC (blue). White arrows point sites in which CD3ζ-mCherry co-localizes with GFP-PRL-1 but not with GFP-PRL-1_ΔCAAX. PRL-1 is indicated in pseudocolored images and CD3ζ-mCherry in the grey scale. Calibration bar is shown. Scale bar 10 μm. Time frame in seconds (s) is indicated. The lower graph shows the quantification of the enrichment of CD3 (red) and PRL-1 (green) in cells showed in upper panels transfected with either GFP-PRL-1 (inverted triangles) or GFP-PRL-1_ΔCAAX (squares) **(c)** In the upper panel, it is shown a frame of the representative time lapse TIRFM experiment played in Movie 4. The green (GFP-PRL-1) and red (CD3ζ-mCherry) channels (grey scale) as well as the merged image is shown. Time of the frame is indicated. The lower panel shows the kymograph obtained in the arrow drawn in the merged image. White arrows in kymograph indicate dynamic co-localization sites. A representative cell of 11 cells tracked in n=3 experiments are shown. (**a** and **b**) 13 conjugates in n=5 experiments were analyzed. The observed distribution of the GFP-PRL-1_ΔCAAX mutant was recorded in three cells.

**
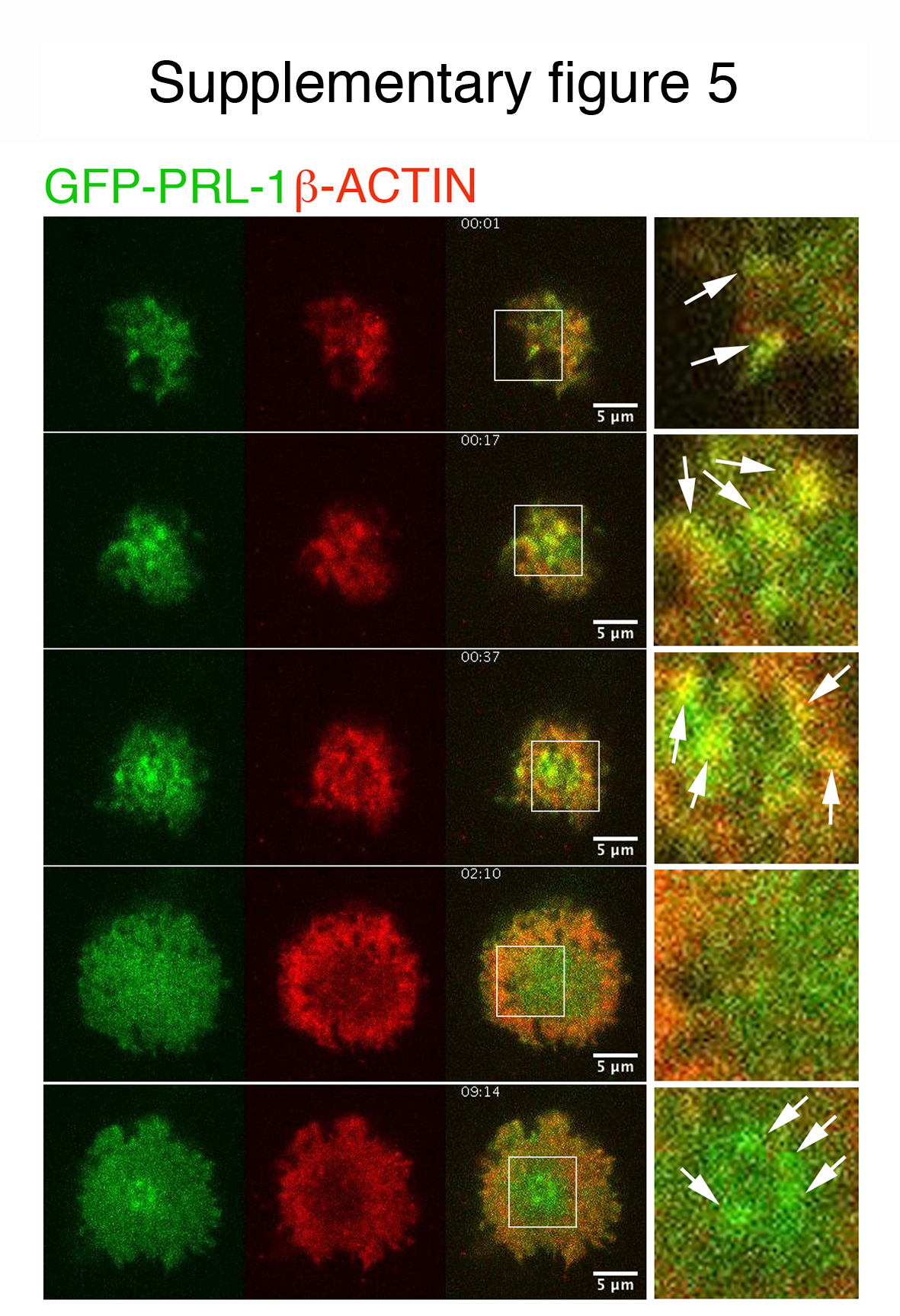
**

**Supplementary Figure 5: Dynamics of GFP-PRL-1 and F-actin at the IS.** Green, red and merged images of the several frames obtained from the representative TIRFM experiment shown in movie 7. Time in minutes:seconds and scale bar are indicated. Arrows in zoom images obtained from squared areas indicate sites of co-localization of PRL-1 and β-actin or, at the time frame 09:14, the location of endosomal PRL-1 polarized to the IS. A representative cell is shown of multiple cells analyzed in Figure 5b.

**­­­
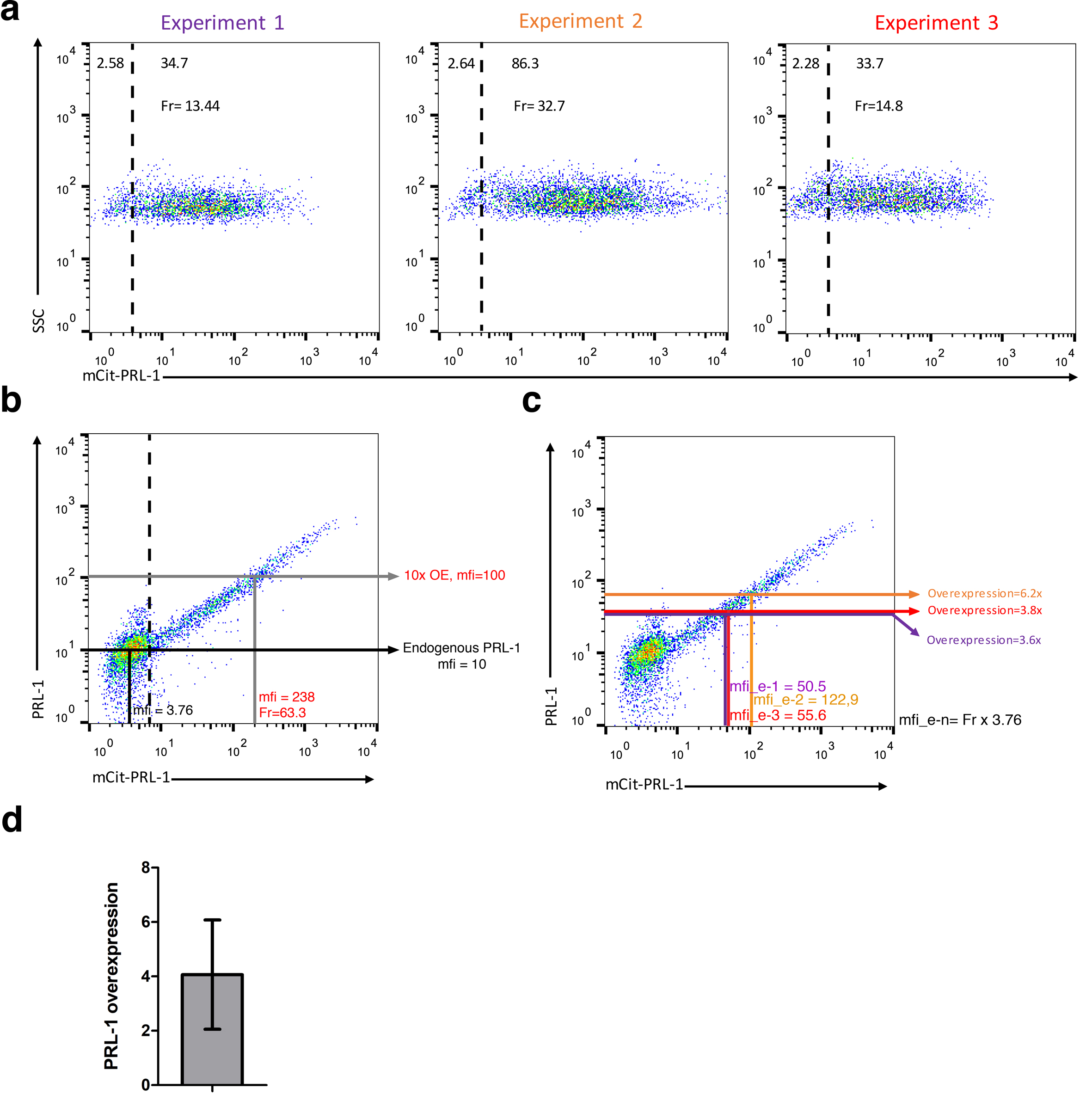
**

**Supplementary Figure 6. Estimation of PRL-1 overexpression.** (**a**) Dot plots of the SSC and mCit-PRL-1 intensity are shown of the three experiments used in figure 6a. Numbers in plots show the mean fluorescence intensity (mfi) of transfected cells and non-transfected cells, separated by dashed lines. The fluorescence ratio (Fr) indicates the ratio between these values. Experiments are color-coded. (**b**) It is shown a dot plot of the staining of PRL-1 (y axis) as a function of the overexpressed (OE) mCit-PRL-1 (x axis). This plot shows the relation between mCit-PRL-1 expression and PRL-1 staining and can be used as a standard line. The mfi is shown for the anti-PRL-1 staining in mCit-PRL-1 negative cells (left side of dashed line, staining of the endogenous PRL-1) and the corresponding mfi in the x-axis (black text). The corresponding mfi in the x-axis and Fr are indicated for a 10-times overexpression of the endogenous PRL-1 (mfi=100) (Fr=63.3=238/3,67) (red text). (**c**) Following this relation in the standard line the mfi corresponding to the Fr of the three experiments in (a) is estimated (mfi_e-n, color-coded). The corresponding values of the mfi for the PRL-1 staining are obtained in the standard line and the overexpression is calculated by normalizing these values with the endogenous levels (mfi=10) (color-coded lines and text). (**d**) The mean ± SD of the PRL-1 OE is represented.


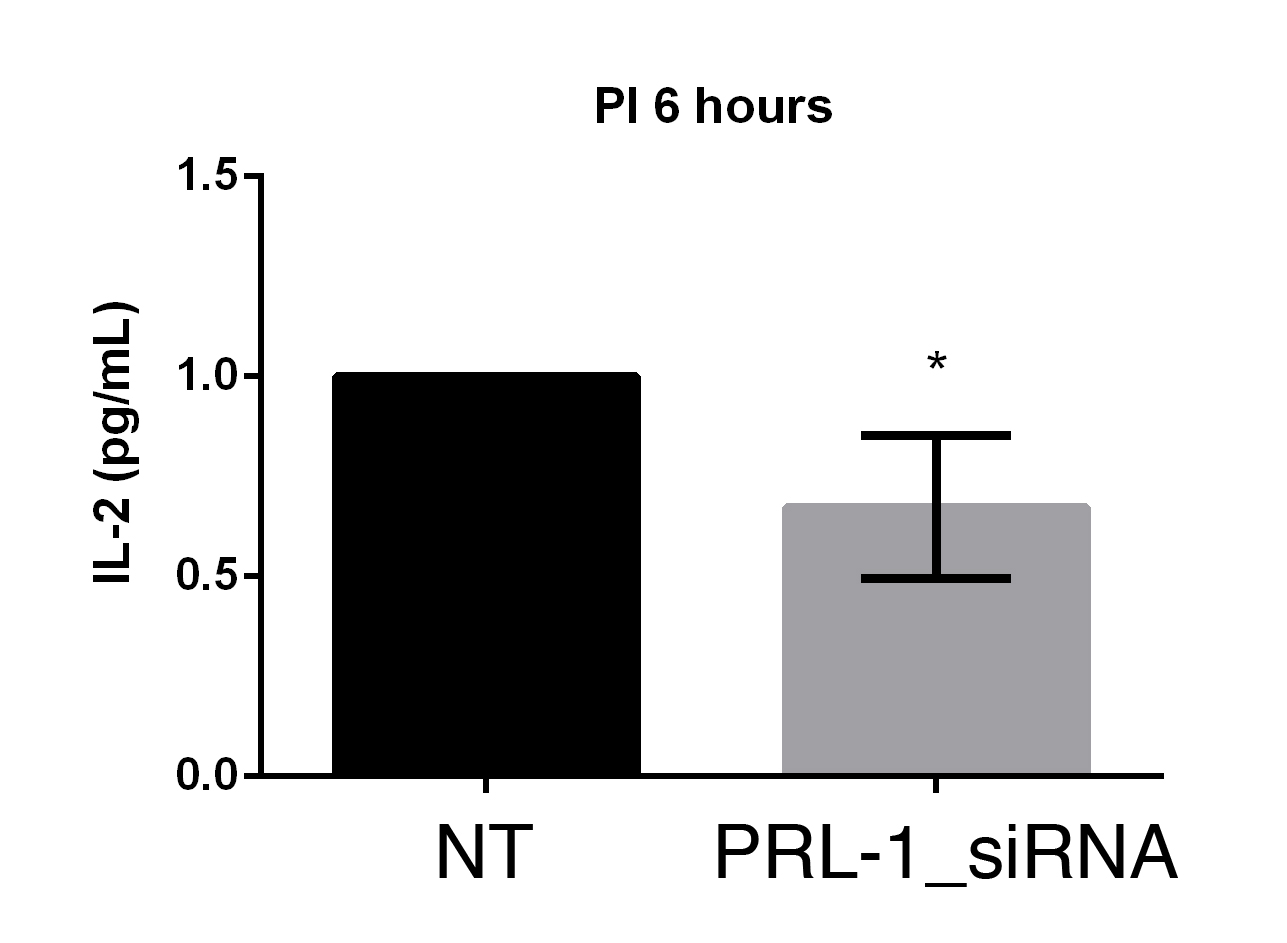


**Supplementary Figure 7. Effect of PRL-1 downmodulation on PI-induced secretion of IL-2**. IL-2 secretion assessed by ELISA in JK cells transfected with the PRL-1 siRNA pool or the NT and stimulated for 6 hours with PMA and ionomycin (PI). It is shown the mean ± SD of the data normalized to the control (NT) in n=4 experiments. PRL-1 siRNA data was compared to one by the one sample t-test. The asterisks represent the p-value: * P≤0.05.

## Movie Legends

**Movie 1 and Movie 2.** **Dynamic distribution of PRL-1 at the IS.** Representative time-lapse confocal microscopy experiments of JK cells cotransfected with CD3ζ-mCherry (red) and GFP-PRL-1 (green) interacting with a SEE-pulsed RAJI cell (blue). In Movie 1 separated channels as well as merged and transmission light images are shown. In Movie 2 merged and transmission light images are shown. Elapsed time 5 seconds (sec) in Movie 1 or 10 sec in Movie 2. Scale bars 10 μm.

**Movie 3 and Movie 4. Dynamic co-localization of PRL-1 and CD3ζ-mCherry at the IS.** Representative time-lapse TIRFM experiments of JK cells cotransfected with CD3ζ-mCherry (red) and GFP-PRL-1 (green). Red (left) and green (middle) channels as well as the merged image (right) are shown. The penetration depth of the TIRF illumination was 150 nm (Movie 3) and 200 nm (Movie 4). Time and scale bar are indicated on the merged image. Time minutes:seconds is labeled. Elapsed time 1 second.

**Movie 5.** **Co-localization of PRL-1 and CD3 during early adhesion of T cells to activating surfaces.** Representative time-lapse TIRFM experiments of JK cells cotransfected with CD3ζ-mCherry (red) and GFP-PRL-1 (green). Green (left, displayed as pseudolocor) and Red (middle) channels as well as the merged image (right) are shown. Penetration depth 150 nm. Time minutes:seconds is indicated on the pseudocolored image and the scale bar on the merged image. Elapsed time 1 second.

**Movie 6. Enrichment of GFP-PRL-1 at sites of actin polymerization during early adhesion of T cells to activating surfaces.** Representative time-lapse TIRFM experiments of JK cells cotransfected with GFP-PRL-1 (green) and pCAGLifeAct–TagRFP (red). Green (left) and red (middle) channels as well as the merged image (right) are shown. Penetration depth 150 nm. Time minutes:seconds and scale bar (10 μm) are indicated. Elapsed time 1 second.

**Movie 7. GFP-PRL-1 and actin dynamics at the IS.** Representative time-lapse TIRFM experiments of JK cells cotransfected with GFP-PRL-1 (green) and mCherry-β-actin (red). Green (left) and red (middle) channels as well as merged images (right) are shown. Penetration depth 150 nm. Time minutes:seconds and scale bar (5 μm) are indicated on the images. Elapsed time 0.5 seconds.
